# Structural Characterization of Polysaccharide Derived from *Gastrodia elata* and Its Immunostimulatory Effect on RAW264.7 Cells

**DOI:** 10.3390/molecules27228059

**Published:** 2022-11-20

**Authors:** Hao Guan, Xi Ling, Juan Xu, Yongquan Zhu, Jiayan Zhang, Xiangyi Liu

**Affiliations:** 1 Key Laboratory of State Forestry and Grassland Administration on Highly-Efficient Utilization of Forestry Biomass Resources in Southwest China, Southwest Forestry University, Kunming 650224, China; 2 College of Architecture and Urban Planning, Kunming University of Science and Technology, Kunming 650050, China; 3 College of Materials and Chemical Engineering, Southwest Forestry University, Kunming 650224, China; 4 Heze Southwest Biotechnology Co., Ltd., Kunming 650506, China

**Keywords:** polysaccharide, structure, immunomodulatory, pathways

## Abstract

A polysaccharide from *Gastrodia elata* (named GEP-1) was isolated with a DEAE-52 column and Sephadex G-100 column. The structural characteristics showed that GEP-1 was mainly composed of glucose (92.04%), galactose (4.79%) and arabinose (2.19%) with a molecular weight of 76.444 kDa. The polydispersity (Mw/Mn) of GEP-1 was 1.25, indicating that the distribution of molar mass (Mw) was relatively narrow, which suggested that GEP-1 was a homogeneous polysaccharide. Moreover, the molecular conformation plot of the root mean square (RMS) radius (<$r_{g}^{2}$> 1/2) versus Mw yielded a line with a slope less than 0.33 (0.15 ± 0.02), displaying that GEP-1 is a compact and curly spherical molecule in NaNO_3_ aqueous solution. NMR and methylation analyses revealed that the main chain structure of GEP-1 was α-(1→4)-glucans. Furthermore, it was proven that GEP-1 possessed cytoproliferative and enhancing phagocytic activities and induced cytokine (TNF-α, IL1-β) and nitric oxide (NO) release in macrophages by upregulating the related gene expression. In addition, the RNA-seq results suggested that the GEP-1-induced immunomodulatory effect was mainly caused by activation of the NF-κB signaling pathway, which was further verified by NF-κB ELISA and pathway inhibition assays. As a result, GEP-1 exhibits the potential to be developed as a novel cheap immunostimulant without obvious toxicity.

## 1. Introduction

*Gastrodia elata* (*G. elata*) belongs to the Orchidaceae family, is a traditional Chinese medicine and has recently been labeled as a healthy food [1]. The main active ingredients of *G. elata* include gastrodin, *G. elata* aglycone, and *G. elata* polysaccharide [2]. It has been reported that *G. elata* exhibits multiple therapeutic neuroactive effects, such as improving memory [3], anti-convulsion [4], preventing senescence [5], neuroprotection [6], etc.

Polysaccharides are a class of biological macromolecules that contain ten or more monosaccharides [7]. Polysaccharides are considered to be potentially bioactive components that exhibit great value for exploitation and have a variety of biological activities, such as immunomodulatory [8,9], antiviral [10,11], antioxidant [12,13], and antitumor [14] activities. In recent years, various studies have been carried out on the composition, physicochemical properties, and biological activities of *G. elata* polysaccharides (GEPs). It was found that GEPs improve the learning memory capacity of D-galactose-induced senescent mice, as well as the activity of oxidative metabolic enzymes in vivo [15]. Liu et al. found that GEPs exhibited a strong antitumor effect on H22-loaded mice and reduced tumor weight [16]. Huo et al. extracted two polysaccharides from *G. elata* that promoted the growth of *Akk. muciniphila* from high-fat diet (HFD) fecal microbiota [17].

The molecular weights of polysaccharides ranging from tens of thousands to millions have been reported, which mainly determines physical properties, such as solubility, stability and viscosity [18]. Polysaccharides with different molecular weights also differ in their bioactivities [19]. Some plant polysaccharides with lower molecular weight exhibit good anti-inflammatory activity by inhibiting the expression of chemokines and adhesion factors, promoting and inducing the production of cytokines [20]. However, there is literature showing that polysaccharides with high molecular weights had better pharmacological effects than those with low molecular weights. Furthermore, some low molecular weight fractions (<10 kDa or 30 kDa) have even been shown to be biologically inactive [21]. That is to say, the molecular weight of a polysaccharide plays an important role in its biological activities.

Macrophages play an important role in both innate and acquired (humoral and cellular) immune responses [22]. Macrophage activation is closely linked to pattern recognition receptors (PRRs) on the macrophage surface, including Toll-like receptors (TLRs), Dectin-1 and complement receptor 3 (CR3). Signaling pathways, such as nuclear factor-kappa B (NF-κB) and mitogen-activated protein kinases (MAPKs), can be activated when the active substance binds to PRRs [23]. Wang et al. found that *Athyrium multidentatum (Doll.) Ching* polysaccharides activate macrophages mainly through MAPK and alternative NF-κB signaling pathways via CD14/TLR4 and Dectin-2 receptors [24]. *Caulerpa chemnitzia* polysaccharides were observed to show an immunomodulatory effect by activating the NF-κB signaling pathway and arachidonic acid metabolism pathway [25]. However, to the best of our knowledge, studies on the immunological activity of GEPs and their molecular mechanisms in macrophages have not been reported.

In the present study, we extracted a neutral homogeneous polysaccharide (named GEP-1) from *Gastrodia elata*, and its structural characteristics and immunostimulatory activity were systematically investigated. Importantly, the possible molecular mechanisms of GEP-1 in the activation of RAW 264.7 macrophages were explored by RNA-seq and verified by pathway analysis. This study aimed to establish an experimental basis for the development and exploitation of GEP-1 as a potential immunomodulator.

## 2. Results

### 2.1. Extraction and Purification of Polysaccharides

*G. elata* powder (400 g) was extracted twice with hot water, and 62.224 g (15% yield) residues were obtained after centrifugation and ethanol precipitation. The residues were processed with Sevag solvent to remove protein and aqueous extracts. The water-soluble refined polysaccharide (10.338 g, 2.58% yield) was obtained from extracts after ethanol precipitation and lyophilization. All refined polysaccharides were separated through a DEAE-Sepharose Fast-Flow column, and 3.57 g of the most abundant fraction (named GEP-D) with a yield of 0.89% was collected from 9 to 15 tubes after lyophilization. GEP-D was further purified with a Sephacryl S-400HR column, and a purified neutral polysaccharide called GEP-1 (2.67 g, 0.67% yield) was obtained after lyophilization.

### 2.2. Monosaccharide Composition and Molecular Weight

**Figure 1:**
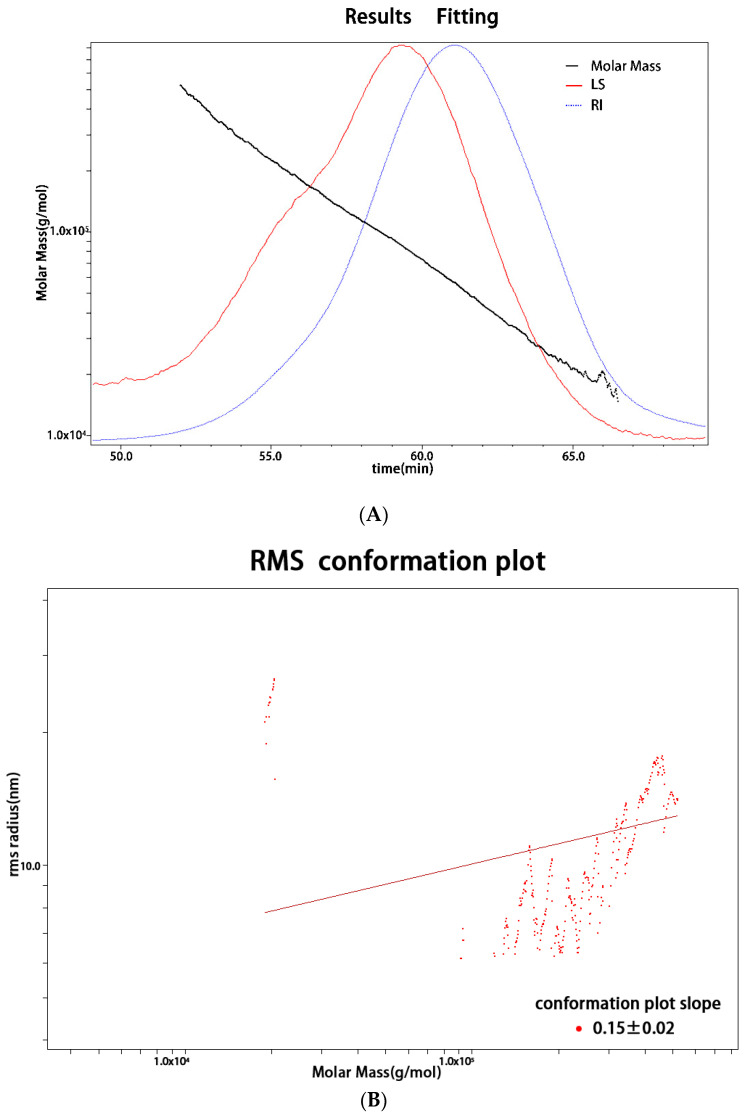

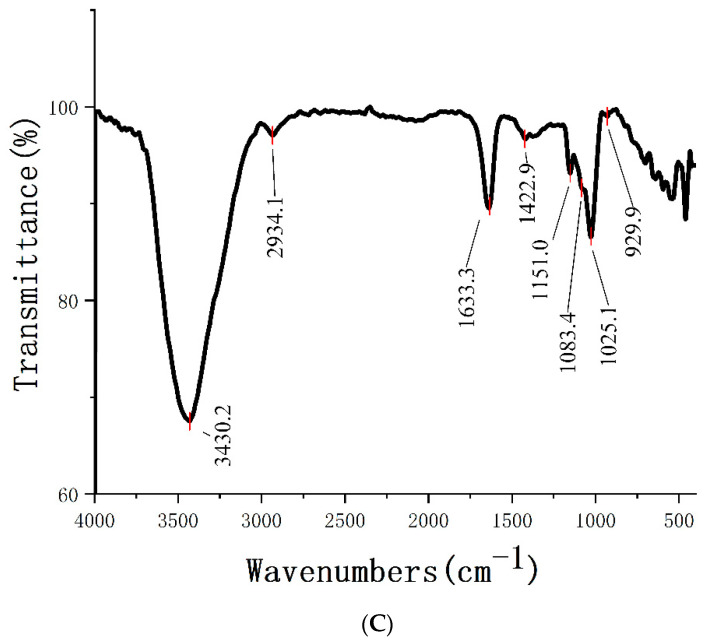
(**A**) Molecular weight determination, (**B**) Log–log plot of *r_g_* versus Mw; RMS—root mean square; (**C**) FT-IR spectrum of GEP-1.

The monosaccharide composition analysis showed that GEP-1 was predominantly composed of glucose (92.04%) and small amounts of arabinose (2.19%) and galactose (4.79%) (Table 1). The molar mass (Mw) of GEP-I was calculated as 76.444 kDa, which was higher than that reported in previous studies [17]. The polydispersity (Mw/Mn) of GEP-1 was 1.25, indicating that the Mw distribution was relatively narrow, which suggested that GEP-1 was a homogeneous polysaccharide (Figure 1A). As shown in Figure 1B, the molecular conformation plot of root mean square (RMS) radius (<$r_{g}^{2}$> ^1/2^) versus Mw yields a line in which the slope is less than 0.33 (0.15 ± 0.02), displaying that GEP-1 is a compact and curly spherical molecule in a NaNO_3_ aqueous solution [26].

### 2.3. FT-IR Analysis

The infrared spectrum of GEP-1 was shown in Figure 1C. The strong and wide absorption peak at 3427.1 cm^−1^ was caused by the stretching vibration of the hydroxyl group (O-H) of polysaccharides. The stretching vibration peak at 1422.9 cm^−1^ and angular vibration peak of 2935.7 cm^−1^ were produced by methylene (-C-H-). There was a strong absorption peak at 1637.9 cm^−1^, which was the stretching vibration of -C=O, indicating the presence of an aldehyde group (-CHO). The absorption peaks at 1151 cm^−1^ and 1085 cm^−1^ represented the existence of -C-O bonds. The absorptions at 1151, 1085, and 1022 cm^−1^ indicated a pyranose form of the glucosyl residue.

### 2.4. Methylation Analysis

The individual peaks and cleavage profiles of GEP-1 were identified by gas chromatography with relative retention times and mass spectra. The percentage content of methylated sugars was expressed as the ratio of peak areas. A total of three main fragment peaks were found by GC-MS (Table 2). Among them, the molar ratio of 1,4,5-tri-O-acetyl-2,3,6-tri-O-methyl galactitol was the highest, which indicated that the polysaccharide was mainly composed of the 1→4-linkage. The presence of 1,5-di-O-acetyl-2,3,4,6-tetra-O-methyl glucitol and 1,4,5,6-tetra-O-acetyl-2,3-di-O-methyl glucitol indicated that the CP consisted of a terminal and 1→4,6-linkage. Moreover, the molar ratio of 1→4-linked, terminal and 1→4,6-linked Glcp was determined to be 13.69:1.26:1. The proportion of side chains in GEP-1 was smaller than that in other reported polysaccharides from *G. elata* [15]. Differences in the type of *G. elata* and the method of preparation of the polysaccharide may be one of the reasons.

### 2.5. NMR Analysis

**Figure 2:**
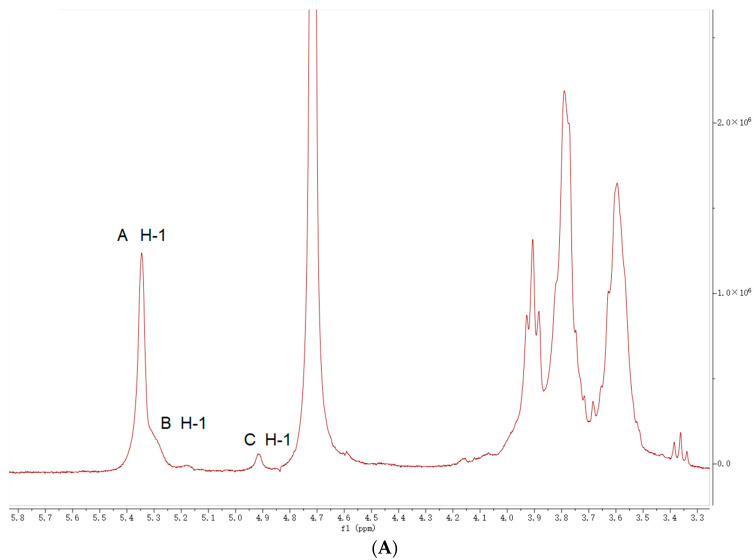

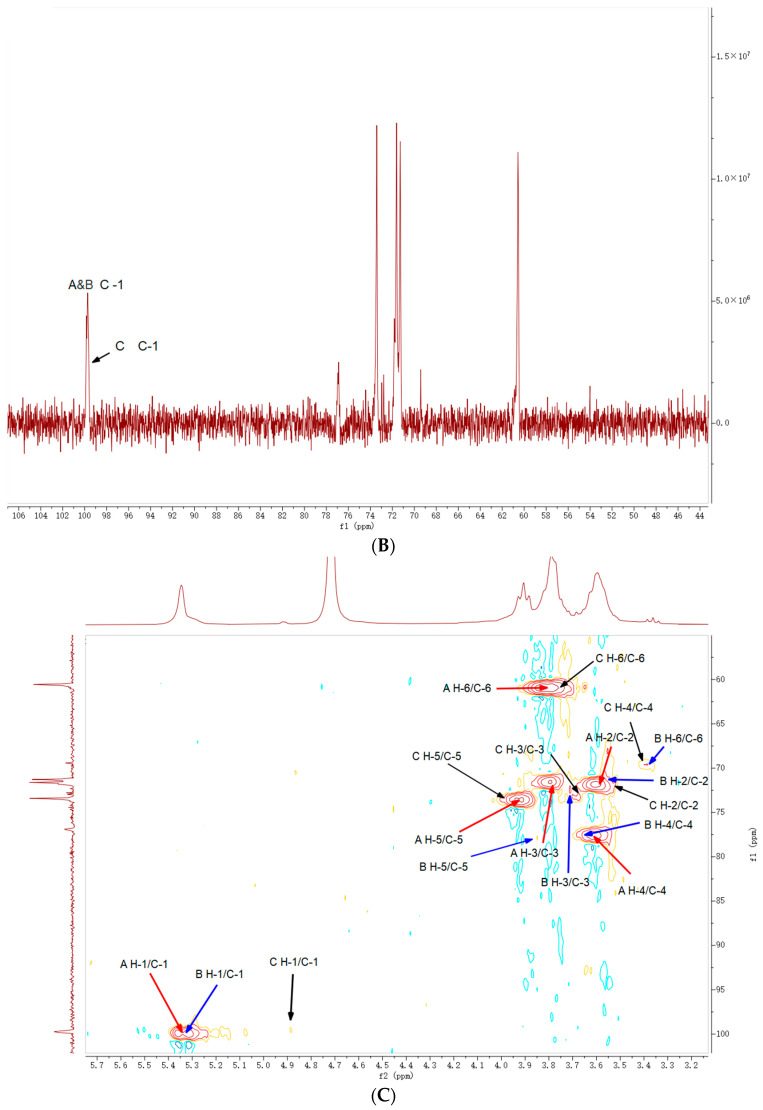

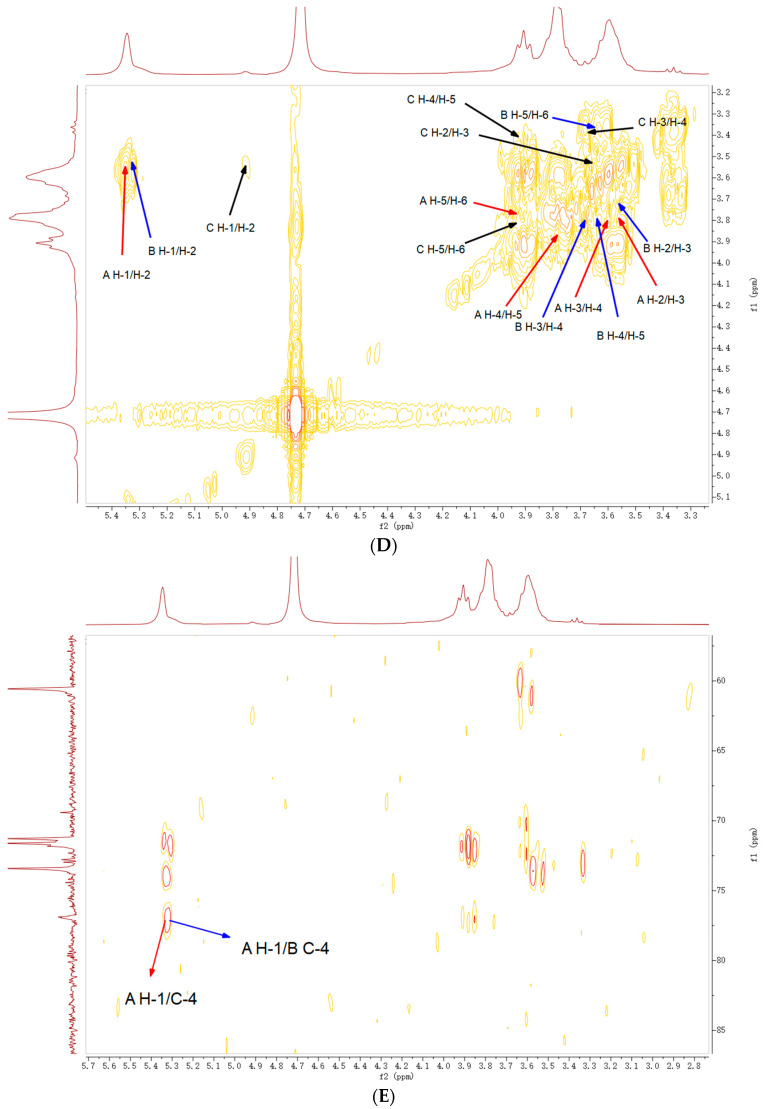
1H (**A**), 13C (**B**), HSQC (**C**), COSY (**D**) and HMBC (**E**) NMR spectra of GEP-1. The letters “A”, “B”, and “C” in the diagram represent residues A, B and C.

As shown in Figure 2A, the ^1^H NMR characteristic signal of GEP-1 was mainly from 3.34 to 5.35 ppm, and the characteristic signal in ^13^C NMR was mainly in the range of 61 ppm to 100 ppm (Figure 2B), which is typical in polysaccharides. Three anomeric proton signals can be found at 5.33, 5.28 and 4.91 ppm in the ^1^H NMR spectrum and are labeled A, B and C, respectively. Combined with the results of the ^13^C NMR spectrum and cross peaks in the HSQC spectrum, the anomeric carbon signal of residue A at 99.75 ppm was correlated to anomeric proton signals at 5.33 ppm. Similarly, the chemical shifts of H-1/C-1 of residues B and C can be marked at 5.32/99.62 and 4.91/99.17 ppm, respectively. Based on a previous study [27], it was suggested that all residues in GEP-1 were present in the α-configuration.

The correlation spectrum (COSY) is a commonly used homonuclear shift correlation spectrum. In general, it reflected the coupling relationship between neighboring hydrocarbons. The proton chemical shifts of residue A with the most intensive signal were observed at 3.58, 3.90, 3.61, 3.79 and 3.80 ppm for H-2, H-3, H-4, H-5 and H-6 from the cross peak in COSY (Figure 2D). After that, the signals at 71.91, 73.69, 77.51, 71.66 and 60.97 ppm were assigned to the C-2, C-3, C-4, C-5, and C-6 of residue A based on the correlations of C/H in the on-anomeric region of the HSQC spectrum (Figure 2C). The downfield chemical shift on C-4 at 77.51 proved that (1→4)-linkages existed in residue A. Thus, residue A was regarded as →4)-α-Glcp-(1→ based on the molar ratio of residues from methylation analysis and NMR data reported in the literature [28].

Likewise, for residue B, the resonance of H-2 was verified at 3.55 ppm through its correlation with H-1 (5.32 ppm) in the COSY. Following the same method in residue A, the chemical shifts at 3.55/71.84, 3.92/73.61, 3.65/77.34, 3.73/71.92 and 3.37/69.88 were attributed to H-2/C-2, H-3/C-3, H-4/C-4, H-5/C-5 and H-6/C-6 in residue B. Because of the downfield shift on both C-4 (77.34 ppm) and C-6 (69.88 ppm), residue B was assigned to →4)-6-α-Glcp-(1→. For residue C, the chemical shifts on H-2/C-2 (3.53/72.71 ppm), H-3/C-3 (3.87/72.92 ppm), H-4/C-4 (3.32/70.24 ppm), H-5/C-5 (3.76/72.37 ppm) and H-6/C-6 (3.81/60.83 ppm) were successfully found in COSY and HSQC. Comparing the proton and carbon chemical shifts with a previous study [24], residue C was assigned to α-D-Glcp-(1→).

As shown in Figure 2E, HMBC experiments were performed to determine the glycosidic linkages between sugar residues. The cross peak between H-1 (5.33 ppm) and C-4 (77.51 ppm) of residue A indicated that the backbone of GEP-1 was →4)-α-Glcp-(1→ connected by (1→4)-O-glycosidic bonds. The cross peak of H-1 (5.28 ppm) in residue A and C-4 (77.51 ppm) of residue B was obtained from HBMC, indicating that →4)-α-Glcp-(1 and →4)-6-α-Glcp-(1→ were linked to each other [29]. The shifts of H-1 to H-6 and C-1 to C-6 for residues A, B, and C are assigned in Table 3.

### 2.6. DSC Analysis

**Figure 3:**
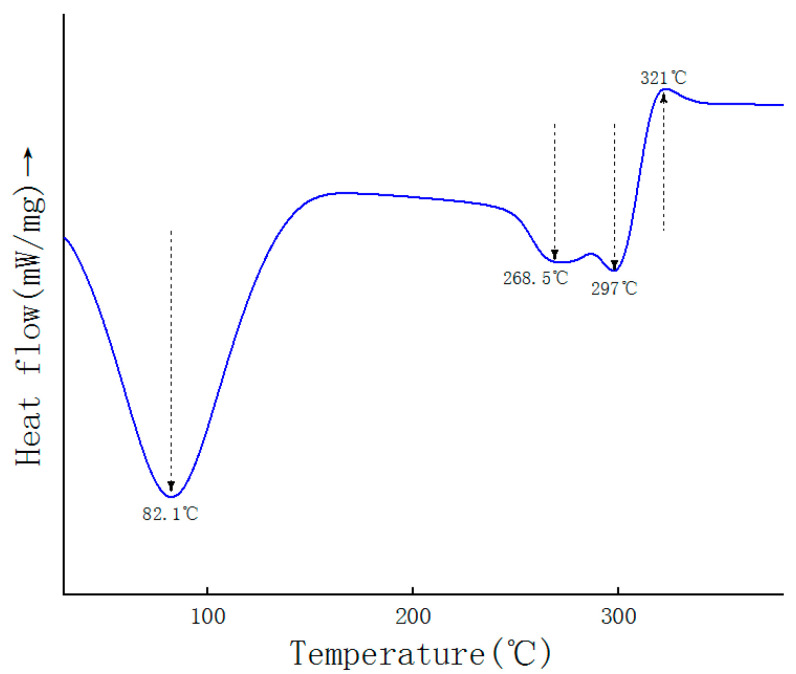
The DSC curve of GEP-1.

DSC represents the thermal effect of the sample when it undergoes physical or chemical changes. DSC could be used to determine the thermal properties, stability and interactions of macromolecules. As shown in Figure 3, GEP-1 had both endothermic and exothermic reactions with increasing temperature. The DSC curve of the first stage showed a broad heat absorption peak at 82.1 °C, which was the process of removing the adsorbed water. In the second stage, the endothermic reaction at 268.5 °C may be due to the elimination of hydroxyl groups from the polysaccharide. A hydroxyl-OH stretching vibration peak at 3427.1 cm^−1^ was also detected in the IR spectrum. In the third stage, The endothermic peak at 288–303 °C is due to the melting of the polysaccharide derivatives at the melting temperature Tm = 297 °C. In the last stage, the exothermic peak near 321 °C, is attributed to the degradation of carbohydrates. It was shown that GEP-1 has good thermal stability and possesses great potential for further development of applications.

### 2.7. Cell Proliferative and Phagocytic Activities

**Figure 4:**
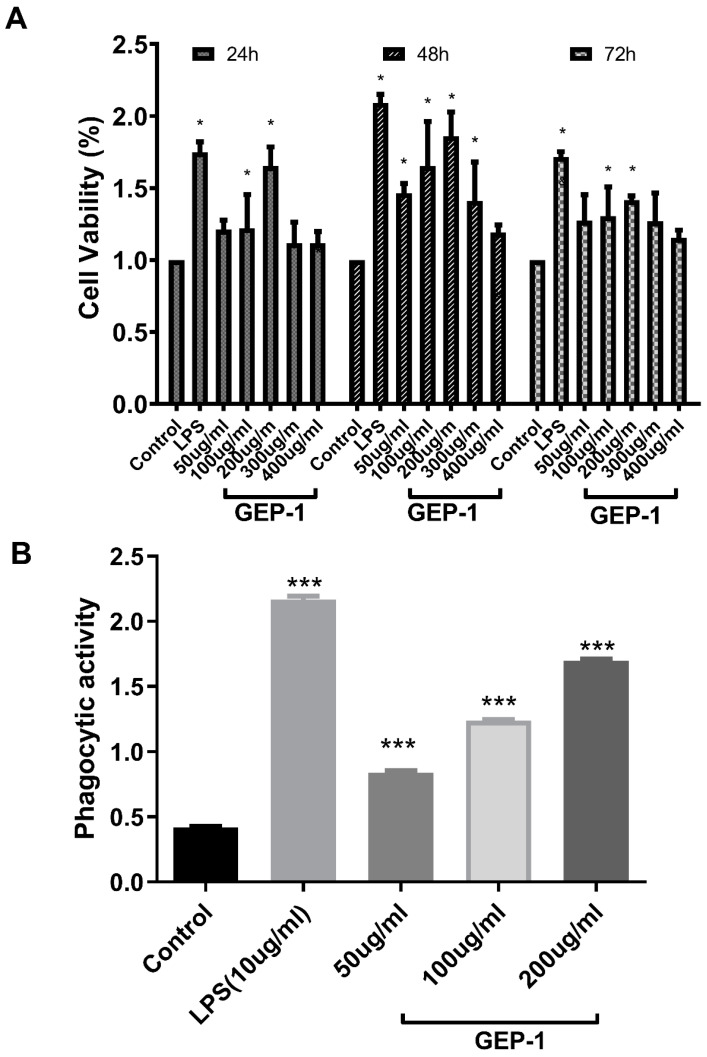
Effects of GEP-1 on cell proliferation (**A**) and phagocytic activities (**B**). Control, cells without treatment; LPS, LPS group, cells treated with 10 μg/mL LPS alone; GEP-1 groups, cells treated with GEP-1 at concentrations ranging from 50 to 400 μg/mL. The symbol “*” represents a significant difference compared with the control group (*p* < 0.05). “***” represents a significant difference compared with the control group (*p* < 0.01).

A cell proliferation assay was performed to detect GEP-1 cytotoxicity at various concentrations and durations in RAW 264.7 cells. As shown in Figure 4A, cell viability was higher in all concentrations of GEP-1 treatment than in the control, and the maximum cell viability was reached at a concentration of 200 μg/mL. Therefore, GEP-1 was almost noncytotoxic to RAW264.7 cells. Moreover, the viability of cells treated with GEP-1 (50–400 μg/mL) for 48 h was higher than those obtained in the other two groups (24 h and 72 h). Obviously, GEP-1 has the same function as LPS in promoting macrophage cell viability. At the same time, a duration of 48 h and concentrations of 50, 100 and 200 μg/mL of GEP-1 was selected for the following experiments.

Figure 4B showed that GEP-1 significantly enhanced phagocytosis of RAW264.7 cells at all concentrations (50, 100, 200 μg/mL) compared to that of the control group. These results revealed that GEP-1 possessed cytoproliferative and enhanced phagocytic activities in macrophages.

### 2.8. Cytokine and NO Levels

**Figure 5:**
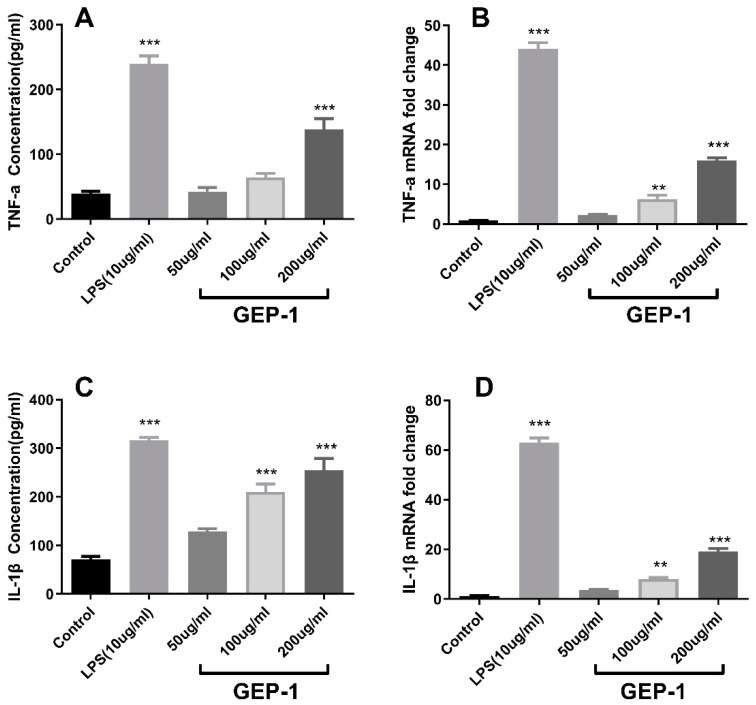

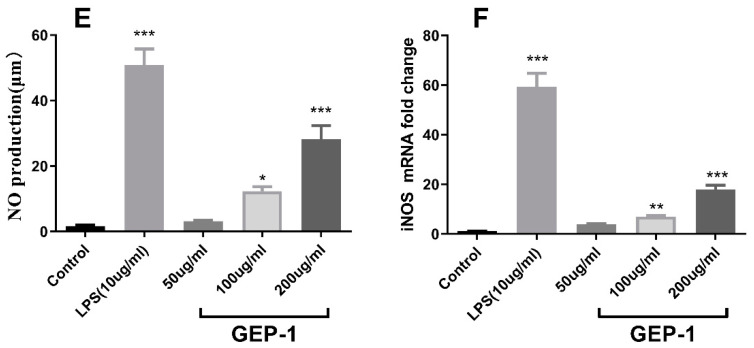
Effects of GEP-1 on the secretion or production of TNF-α (**A**), IL-1β (**B**) and NO (**C**). Effects of GEP-1 on the gene expression levels of TNF-α (**D**), IL-1β (**E**) and iNOS (**F**) * denotes the differences between the control group and other groups, * *p* < 0.05, ** *p* < 0.01, and *** *p* < 0.001.

In this study, the protein levels of TNF-α and IL-1β were measured by ELISA (Figure 5A,B), and their gene expression levels were evaluated by qPCR (Figure 5D,E). As presented in the figure, the doses of GEP-1 at 50 and 100 µg/mL showed a nonsignificant effect on TNF-α secretion compared to that of the controls, while high doses of GEP (200 µg/mL) exhibited a highly significant effect on TNF-α secretion (*p* < 0.01). At the gene expression level, a high dose of GEP-1 also significantly upregulated the expression of the TNF-α gene. Likewise, IL-1β secretion by macrophages also increased dramatically (*p* < 0.01) in both the medium- and high-dose GEP-1-treated groups (100 and 200 µg/mL) compared to the control group. The gene expression of IL-1β was increased at all three doses, and the high-dose group exhibited a significant increase compared to that of the control group (*p* < 0.01). These results suggested that GEP-1 played an immunoregulatory role by stimulating cytokine secretion via the upregulation of gene expression.

NO production was measured after treating macrophages with different concentrations of GEP. As shown in Figure 5C, both 100 and 200 µg/mL concentrations of GEP showed a significant positive effect on NO production in macrophages compared to that of the control group (*p* < 0.01). Furthermore, the relative effect of GEP-1 on NO production was consistent and linear with dose. On the other hand, inducible NO synthase (iNOS) is responsible for NO generation; therefore, its gene expression levels were measured using qPCR. Figure 5F shows the significant promotion effects of medium and high doses of GEP-1 on iNOS gene expression. These results revealed that GEP-1 could upregulate iNOS gene expression to synthesize NO, thereby exerting an immunoregulatory effect.

### 2.9. Transcriptomic Analysis

**Figure 6:**
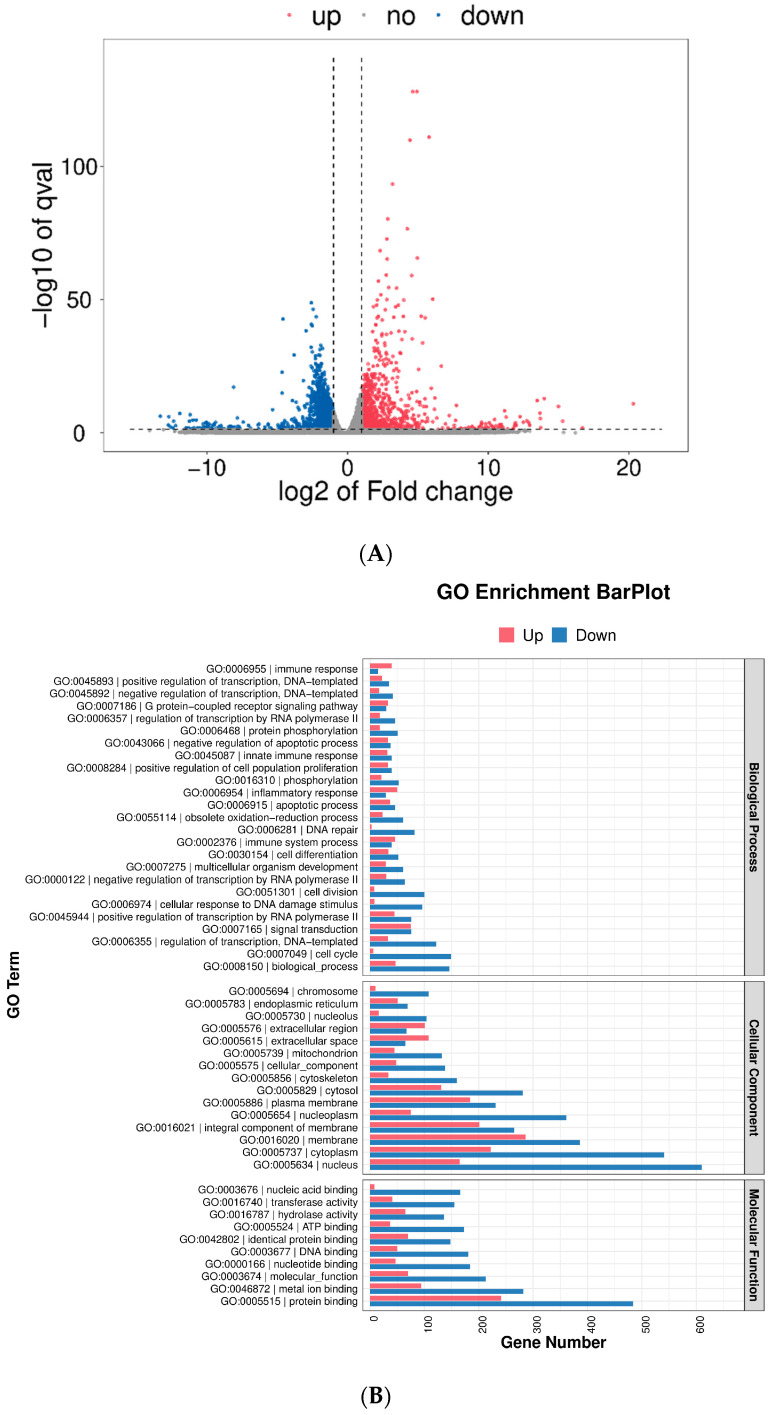

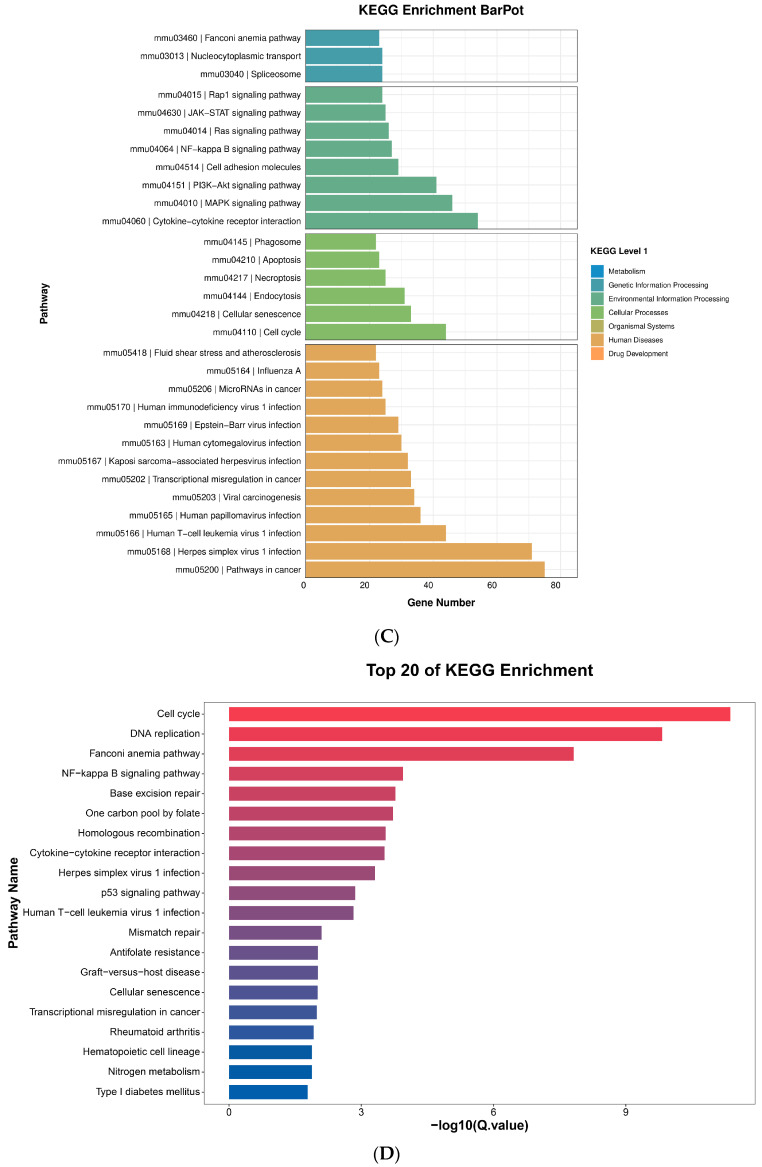

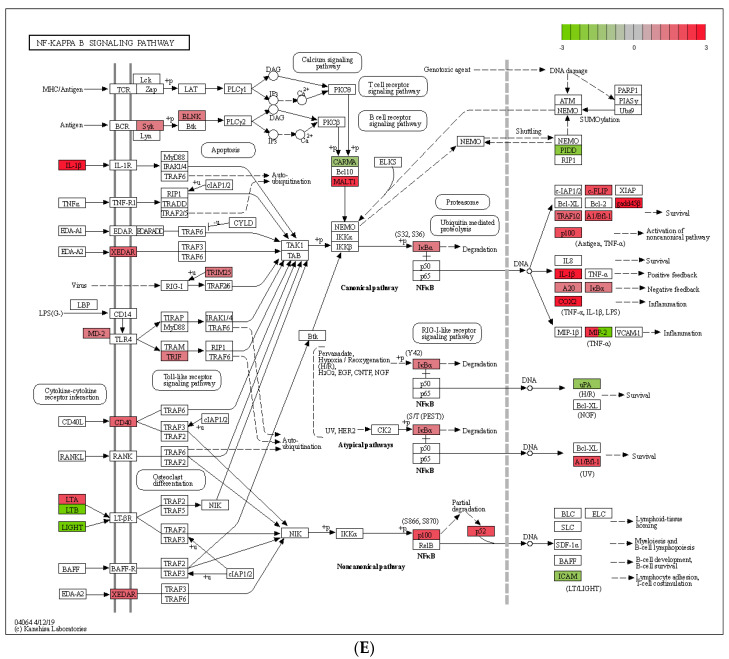
Effects of GEP-1 on gene changes in RAW 264.7 macrophage cells compared with the normal group (NG). (**A**), volcano plots of RAW 264.7 cells; (**B**), GO enrichment analysis of DEGs; (**C**), KEGG analysis of DEGs; (**D**), Top 20 enriched KEGG pathways. (**E**), DEGs in the NF-κB signaling pathway from RNA-seq.

In this study, RAW264.7 cells treated with GEP-1 were assayed using RNA-seq technology to analyze the effect of GEP-1 on gene regulation in macrophages. As shown in the volcano plots (Figure 6A), a total of 2564 differentially expressed genes (DEGs) were identified in the treatment group (200 µg/mL GEP-1) compared with the control group (NG), including 898 upregulated genes and 1666 downregulated genes.

Gene Ontology (GO) is an internationally standardized gene function classification system that provides a dynamically updated set of controlled vocabulary to comprehensively describe the properties of genes and gene products in an organism [30]. The GO enrichment analysis was described mainly in terms of the molecular function (MF), the cellular component (CC), and the biological process (BP). Many DEGs appeared in the CC and MF categories (Figure 6B). “Nucleus”, “cytoplasm” and “membrane” were the top three significant terms in the CC part, and the most significant terms in the MF categories were in “protein binding”. On the other hand, the terms in BP suggested that GEP-1 was involved in cell proliferation and the immune response, exerting an immunomodulatory effect.

The KEGG database is a collection of databases and accompanying software for using genome information to understand and simulate higher-order functional, cellular, or organismal behavior [31]. The results indicated that multiple immune-related pathways are involved in processing genetic information (Figure 6C), such as the NF-κB, MAPK and JAK/STAT signaling pathways. Importantly, the NF-κB signaling pathway was listed in the top 20 KEGG enrichment pathways (Figure 6D). Combined with other literature reports [32], the immune effects of GEP-1 may be mainly related to the activation of the NF-κB signaling pathway.

### 2.10. Pathway Analysis

#### 2.10.1. NF-κB Assay

**Figure 7:**
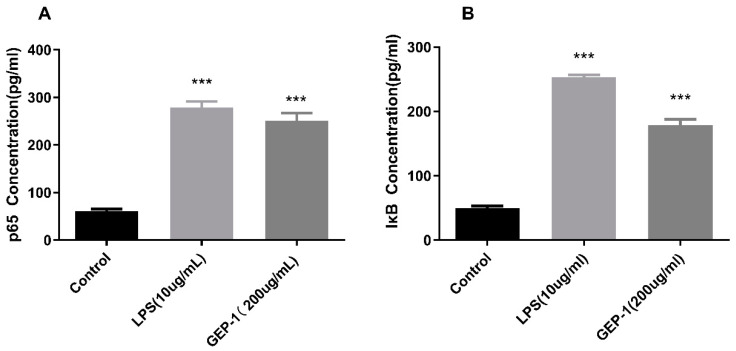
Effects of GEP-1 on p65 (NF-κB) (**A**) and IκB (**B**) protein expression in RAW264.7 cells. *** denotes the differences between the control group and other groups, *** *p* < 0.001.

From the RNA-seq results, the gene expression of NF-κB was upregulated (Figure 6E), and the NF-κB pathway was listed in the top 20 enrichment pathways. Hence, the protein levels of NF-κB and IκB were measured by a rat ELISA kit to verify the essential role of this pathway. As shown in Figure 7A,B, GEP-1 showed a noticeable increase in NF-κB activation compared with that in the control group (*p* < 0.01).

#### 2.10.2. NF-κB Pathway Inhibition Assay

**Figure 8:**
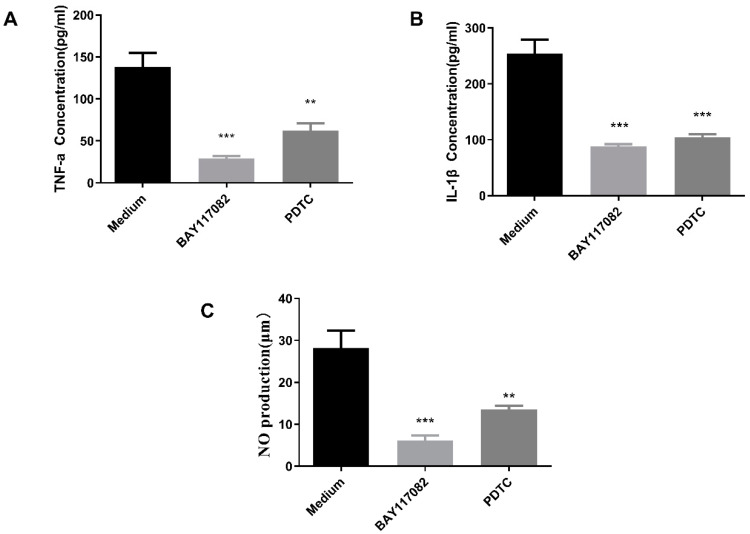
Effects of NF-κB inhibitors on TNF-α (**A**), IL-1β (**B**) and NO (**C**) production in GEP-1-treated RAW264.7 cells. The macrophages were treated with GEP-1 in the absence or presence of specific pathway inhibitors, and cytokine and NO production in the culture supernatants was determined. * denotes the differences between the control group and other groups, ** *p* < 0.01, and *** *p* < 0.001.

To establish the relationship between cytokine release and the NF-κB pathway, the effects of PDTC (NF-κB inhibitor) and BAY117082 (IκB inhibitor) on GEP-1-induced cytokine secretion and NO production were determined. As illustrated in Figure 8A–C, both specific inhibitors significantly suppressed TNF-α, IL-1β and NO production in GEP-1-treated RAW264.7 cells compared with the medium group (*p* < 0.01). In summary, these results demonstrated that NF-κB pathways were involved in the effect of GEP-1 stimulation on NO and cytokine release by macrophages.

## 3. Discussion

In the current study, a purified polysaccharide (GEP-1) with a molecular weight of 76.444 kDa was isolated from *Gastrodia elata* powder. GEP-1 was mainly composed of glucose (92.04%), galactose (4.79%) and arabinose (2.19%) with the main chain structure α-(1→4)-glucans. Cytoproliferative and enhancing phagocytic activities were found on macrophages with the CCK-8 kit and neutral red assay, and further experiments demonstrated that GEP-1 could upregulate the gene expression of cytokines (TNF-α, IL-1β) and iNOS to increase their secretion in cells. Moreover, 2564 DEGs were observed in RNA-seq, including 898 upregulated genes and 1666 downregulated genes. GO enrichment and KEGG pathway analyses suggested that the immune response induced by GEP-1 was mainly caused by the activation of the NF-κB signaling pathway. Finally, the NF-κB assay and pathway inhibition assay further verified the results of RNA-seq.

*Gastrodia elata* is a traditional Chinese herb that is cultivated in various areas of China, among which Zhaotong species in Yunnan, China, exhibit the best medicinal effect [33]. In the present study, both *Gastrodia* seeds and *Armillaria mellea* were obtained from Zhaotong, and the cultivation method was underwood planting. The polysaccharide content (23%) of our *Gastrodia elata* was higher than that of the previous Zhaotong species, so this study was conducted to analyze its structure and activity. According to previous studies on Zhaotong *Gastrodia elata* polysaccharides [17], both GEP-3 found by Huo et al. and GEP-1 in this study belong to α-(1→4)-glucans. The difference is that the molecular weight of GEP-3 is 20 kDa, while the molecular weight of GEP-1 reaches 76 kDa, indicating that GEP-1a exhibits a higher degree of polymerization. The molecular weight of polysaccharides plays an important role in the activity. In studies by Apostolova et al. and Wu et al., low-molecular-weight fucoidan (<50 kDa) exhibited higher immunological activity than that of high-molecular-weight fucoidan (>100 kDa) [28,29]. In addition, the substitution degree of polysaccharide branched chains also plays a key role in activity [34]. GEP-1 showed a smaller substitution degree than GEP-3, which also suggests that there may be a gap in their activity.

The presence of arabinose and galactose was detected in both monosaccharide fraction analysis and methylation analysis, but the low content of these two monosaccharides and the low solubility of GEP-1 in D_2_O led to a failure in identifying the linkage of these two monosaccharides in GEP-1 through NMR analysis. The methylation analysis suggested that arabinose existed as 1-Ara. Galactose may exist in three linkages, including 1,4-Gal, 1,2,4-Gal and 1,3,6-Gal.

Lipopolysaccharide (LPS) is widely found in gram-negative bacteria and is a macromolecular substance composed of lipids and polysaccharides, also known as an endotoxin [35]. It stimulates a nonspecific immune response in the body and is also an exogenous pyrogen (fever-inducing substance). The polysaccharide component of LPS is an important compound used by the body to recognize foreign bacteria and to initiate innate immunity [36]. Therefore, natural plant polysaccharides are thought to be recognized by the receptor and trigger nonspecific immunity due to their structural similarity to the polysaccharide in LPS. In this study, GEP-1 promoted phagocytic activity and cytokine and NO release from macrophages and showed no toxic effects, suggesting that GEP-1 could be developed as a novel immunostimulant.

In addition, GEP-1 is highly similar in primary structure to amylopectin (α-D-(1 → 4) linear chains with α-D-(1 → 6) branches); however, the literature reports that starch has no positive effect on NO production and TNF-α secretion in macrophages [34]. This may be closely related to their specific spatial structure [37]. The preliminary understanding of the molecular chain conformation of GEP-1 in the log–log plot of r_g_ versus Mw indicates that GEP-1 has a near-spherical conformation. The relationship between this conformation and activity needs to be further investigated. Interestingly, almost all reported α-D-glucans that possess immune activity had (1 → 6) branches, which also demonstrated that the substitution of branched chains of polysaccharides exhibited an important effect on their activity.

In recent years, the study of the mechanism of polysaccharide immunoreactivity has increased, and a large number of experiments have shown that the immunoreactivity is associated with gene upregulation of the NF-κB and mitogen-activated protein kinase (MAPK) pathways [38,39,40]. NF-κB is an inducible cytoplasmic transcription factor that plays important roles in regulating multitudinous genes related to inflammation, immune responses and cell survival [41]. It includes classical and alternative pathways. The former releases P50 and p65 through phosphorylation and ubiquitination of IκB proteins, thereby signaling into the nucleus to exert immunomodulatory effects. The alternative pathway involves NIK and IKK-alpha-mediated p100 phosphorylation and processing to p52, resulting in nuclear translocation of p52/RelB heterodimers. In this study, RNA-seq was used to narrow the scope of pathways, and the NF-κB signaling pathway was regarded as a key point for GEP-1 to exert immune activity based on GO enrichment analysis and KEGG analysis. To verify the results, NF-κB (p65) and IκB ELISAs were performed. NF-κB inhibitors were used to demonstrate the relevance of this pathway to the release of cytokines and NO by macrophages. The results showed that both p65 and IκB expression were increased in the GEP-1-treated cells; NF-κB inhibitor decreased the levels of cytokines and NO release. In summary, we identified the NF-κB pathway as an essential signaling pathway for GEP-1 to exert immunomodulatory effects.

Based on other reports of α-(1→4)-glucans, another important signaling pathway is the MAPK [42], which can activate downstream cytosolic proteins and nuclear transcription factors, such as NF-κB and AP-1 [43]. Moreover, Bruce Beutler demonstrated that Toll-like receptor 4 (TLR4) is the LPS receptor and was awarded the 2011 Nobel Prize in Physiology or Medicine [41]. TLR4 has become a hot topic in the recognition of polysaccharides [44,45,46]. For example, Zhang et al. found that polysaccharide TS2–2A isolated from *Trametes sanguinea* Lloyd was recognized by Toll-like receptor 4 (TLR4) to release related cytokines and contributed to immune-enhancing effects [47]. Therefore, further studies will focus on the MAPK and TLR4 pathways to refine the mechanism of GEP-1 immunoreactivity.

## 4. Materials and Method

### 4.1. Materials and Reagents

*Gastrodia elata* were collected from Yunnan Province of China. All the samples were authenticated by prof. Xiangyi Liu in Southwest Forestry University, China. The materials were washed, sliced, air-dried, and crushed into a powder before extraction. DEAE-cellulose 52 and Sephadex G-100 were acquired from Sunresin Co., Ltd. (Xian, China). Lipopolysaccharide (LPS) was obtained from Sigma Chemical Co., Ltd. (St. Louis, MO, USA). Dulbecco’s modified eagle medium (DMEM) with 10% FBS and 1% penicillin-streptomycin and phosphate buffered saline (PBS) from Hyclone Laboratories Inc. Cell counting kit-8 (CCK-8) was purchased from Wuhan Fine Biotech Co., Ltd. (Wuhan, China). Nitric oxide (NO) and mouse ELISA kits were from Sigma Chemical Co., Ltd. (St. Louis, MO, USA). All inhibitors were purchased from Selleck Chemicals (Houston, TX, USA). Quantitative Real-time PCR was acquired from Applied Biosystems, Inc. (Carlsbad, CA, USA). SYBR Mix was provided by BeiJing LABSELECT Co., Ltd. (Beijing, China). Gene primers were prepared by Jinan Ruizhi Biotech. Co., Ltd. (Jinan, China). Chloroform, isopropanol and other chemicals were attained from iNational Pharmaceutical Group Co., Ltd. (Shanghai, China). 

### 4.2. Extraction and Purification of Polysaccharide from G. elata

The polysaccharide was extracted as in a previous study [48]. In brief, the *Gastrodia elata* powder was extracted with deionized water (1: 20, *w*/*v*) at 60 °C in the motor agitator. The extract was centrifuged (8000 r/min) for 15 min and then filtered with slow quantitative filter paper. The extract was concentrated by rotary evaporation and precipitated overnight with 95% ethanol at 4 °C. Sevag solution was added repeatedly to remove protein and the supernatant was dialyzed with a 7000 DA dialysis bag for 72 h and lyophilized as crude polysaccharides.

The DEAE-52 cellulose column was used to elute crude polysaccharides with deionized water at a flow rate of 1 mL/min. The eluted aliquots were collected at 15.00 mL intervals and the phenol-sulfuric acid method was used to monitor the elution. The polysaccharide fractions were dialyzed against tap water for 48 h and distilled water for 24 h using 7000 Da MW cutoff dialysis bags and then lyophilized by a freeze dryer.

The most abundant polysaccharide fraction from DEAE-52 was purified further on a Sephadex G-100 column (2 cm × 60 cm) and eluted with distilled water at a flow rate of 1 mL/min. The eluted aliquots were collected at 5.00 mL intervals and the polysaccharides in the desorption solution were quantified using the phenol-sulfuric acid technique at 490 nm. The GEP-1 was collected after concentration, dialysis and lyophilization. 

### 4.3. Structure Characterization

#### 4.3.1. Determination of Molecular Weight 

The homogeneity and molecular weight of various fractions were determined using Size-Exclusion-Chromatography (SEC)-Multi-Angle Laser Light Scattering (MALLS)-Refractive Index (RI) system [49]. The weight- and number-average molecular weight (Mw and Mn) and polydispersity index (Mw/Mn) of various fractions in 0.1 M NaNO_3_ aqueous solution containing 0.02 percent NaNO_3_ were measured on a DAWN HELEOS-II laser photometer (He-Ne laser, λ = 663.7 nm, Wyatt Technology Co., Santa Barbara, CA, USA) equipped with Three tandem columns (300 × 8 mm, Shodex OH-pak SB-805, 804 and 803; Showa Denko K.K., Tokyo, Japan), which was held at 45 °C using a model column heater. The flow rate is 0.4 mL/min. A differential refractive index detector (Optilab T-rEX, Wyatt Technology Co., Santa Barbara, CA, USA) was simultaneously linked to give the concentration of fractions and the dn/dc value. The dn/dc value of the fractions in 0.1 M NaNO_3_ aqueous solution containing 0.02 percent NaNO_3_ was determined to be 0.141 mL/g.

#### 4.3.2. Determination of Monosaccharide Composition

The monosaccharide compositions of GEP were analyzed by high-performance anion-exchange chromatography (HPAEC) on a CarboPac PA-20 anion-exchange column (3 by 150 mm; Dionex) using a pulsed amperometric detector (PAD; Dionex ICS 5000 system) [48]. Briefly, 5 mg of the sample was hydrolyzed with 2 M trifluoroacetic acid at 121 °C for 2 h. The sample was dried with nitrogen and blow dried after adding methanol to the wash, then the methanol and wash were repeated two to three times. For measurement, the residue was redissolved in deionized water and filtered through a 0.22 m microporous filtering sheet. Fucose, rhamnose, arabinose, galactose, glucose, xylose mannose, fructose, ribose, galacturonic acid, glucuronic acid and mannuronic acid were chosen as standards.

#### 4.3.3. Fourier Transform Infrared Spectroscopy (FT-IR)

The GEP-1 was evenly mixed with KBr and pressed. The infrared spectrum of the sample was obtained by FT-IR instrument (ALPHA-T, BRUKER Co., Billerica, MA, USA) in the range of 400–4000 cm^−1^.

#### 4.3.4. Methylation Analysis

Methylation analysis was performed in accordance with the previous study [50]. 10 mg purified GEP-1 was dissolved in 1 mL of distilled water and reacted with 1 mL of N-cyclohexyl-N-[2-(4-methyl-1-oxa-4-azoniacyclohex-4-yl) ethylenediimino-4-methylbenzenesulphonic acid (100 mg/mL) for 2 h, followed by the addition of 1 mL of imidazole (2 mol/L). The reaction product was divided into two parts. Each part was reacted with NaBH_4_ (30 mg) or NaBD_4_ (30 mg) for 3 h. The reaction was terminated with 0.1 mL of acetic acid. The samples were dialyzed in distilled water for 48 h, freeze-dried and dissolved in 0.5 mL of dimethyl sulfoxide and reacted with 1 mg of sodium hydroxide for 30 min. At the end of the treatment, 0.05 mL of methyl iodide was added and reacted in the dark at 25 °C for 1 h. The mixture was then extracted with CH_2_Cl_2_ and the resulting methylated products were hydrolyzed with 2 mol/L TFA at 121 °C for 90 min. The hydrolysate was reacted with NaBD_4_ (50 μL, 1 mol/L) and ammonium hydroxide (50 μL, 2 mol/L) at room temperature for 2.5 h. The acetylation reaction was then carried out with acetic anhydride (0.25 mL) at 100 °C for 2.5 h. Finally, the methylate alditol acetates were extracted with 0.5 mL of CH_2_Cl_2_ and analyzed on a GC-MS system (Agilent Technologies, Santa Clara, CA, USA). The initial column temperature was maintained at 140 °C for 2 min and then increased at a slope of 3 °C /min to 230 °C. The mass scan range was 30.0 *m/z* to 600.0 *m/z* and partially methylated sugar alcohol acetate was identified based on the mass spectra and relative retention times of the peaks.

#### 4.3.5. NMR Analysis

Twenty milligrams of GEP were dissolved in 0.5 mL D_2_O (99.9%). NMR spectra (^1^H NMR and ^13^C NMR) were recorded at 25 °C on an AVANCEIII NMR 500 spectrometer. Two-dimensional experiments, including the Homonuclear 1H/1H Correlation Spectroscopy (COSY), Heteronuclear Single-quantum Coherence (HSQC), and Heteronuclear Multiple-bond Correlation (HMBC) were also conducted for further analysis.

#### 4.3.6. Differential Scanning Calorimetry (DSC) Analysis

The thermal characteristics of GEP-1 were determined by DSC (NETZSCH-Gerätebau GmbH, Selb, Germany) according to the method of Wang et al. [51]. A sample of 6 mg polysaccharide was accurately weighed and added to the sample tray. During the analysis, the temperature range was set to 25–400 °C with a 10 °C/min heating rate.

### 4.4. Immunomodulatory Activity

#### 4.4.1. Cell Culture

The macrophage RAW264.7 cells were cultured in DMEM supplemented with a mixture of 10%(*v/v*) FBS and 1%(*v/v*) penicillin-streptomycin in an incubator at 37 °C with 5% CO_2_. Treatments included a control group (CG), lipopolysaccharide group (LPS) and various concentrations of GEP-1. For most experiments, cells were allowed to adhere for 24 h before treatment.

#### 4.4.2. Cell Proliferation Assay 

The proliferation of cells was assessed by CCK-8 assay according to the manufacturer’s instructions. Briefly, cells were diluted to 1 × 10^4^ cells/well in 96-well plates and incubated at 37 °C for 24 h in a 5% CO_2_ incubator. Different doses of polysaccharide samples (50/100/200/300/400 μg/mL) and LPS (10 μg/mL) were added and incubated at 37 °C for various times at 24, 48 and 72 h. At the end of the treatment, 10 μL CCK-8 reagent was added and incubated for 2 h at 37 °C. After incubation, the absorbance at 450 nm was measured using a multifunctional microplate meter.

#### 4.4.3. Phagocytic Activity Assay

The phagocytic activity of the cells was estimated by the neutral red uptake method. Briefly, RAW264.7 cells were seeded in 96-well plates at a density of 1 × 10^4^ cells/well and incubated in a 5% CO_2_ incubator at 37 °C for 24 h. Polysaccharide samples at different final concentrations (50, 100, 200 μg/mL) and LPS as positive controls (10 μg/mL) were then added; 12 h later, cells were washed twice with PBS and incubated with 100 μL neutral red solution (0.05%) for 30 min. Additional neutral red was then removed with 0.01 M PBS solution. Cells were lysed by adding 100 μL of glacial acetic acid-anhydrous ethanol (1:1, *v/v*). Finally, the absorbance at 540 nm of samples was detected on a microplate reader.

#### 4.4.4. Assay of Cytokine Secretion

The concentration of RAW 264.7 cells was diluted to 4 × 10^4^ cells/ well in 6-well plates and allowed to adhere for 24 h at 37 °C. After pretreatment with the GEP-1 samples (50, 100, 200 ug/mL) or LPS (1 μg/mL) for 48 h, about 1.4 mL of supernatants were collected and stored at −80 °C. The levels of cytokines including TNF-α and IL-1β secretion in the macrophage culture were estimated using a rat enzyme-linked immunosorbent assay (ELISA) kit.

#### 4.4.5. Assay of NO Production 

RAW264.7 cells (4 × 10^4^ cells) were cultured by adding different concentrations of GCP solutions (50, 100, 200 μg/mL) or LPS (1 μg/mL) for 48 h. The level of NO production was determined by the Griess reagent according to the manufacturer’s instructions.

#### 4.4.6. RT-qPCR Assay of Cytokine and NO

The mRNA expression of TNFα, IL-1β and iNOS was analyzed by quantitative real-time reverse-transcription (qPCR) by a previously standardized protocol [52]. In 6-well plates, cells were diluted to 4 × 10^5^ cells per well. The total RNA of cells was extracted with Trizol reagent according to the supplier’s instructions after treatment with polysaccharide samples (50, 100, 200 μg/mL) and LPS (1 μg/mL) for 48 h. The purity of RNA was evaluated by A260/A280 ratio using a Nano Photometer (P300, Implen, Germany). RT-qPCR was carried out on a Light cycler 480 II PCRs instrument (Roche) to value mRNA levels. The cycling conditions were 95 °C for 30 s, 40 cycles at 95 °C for 5 s, 55 °C for 20 s, and 72 °C for 30 s. GAPDH was used as a standard against which other RNA values may be compared. 2^−∆∆Ct^ was used to indicate the gene expression values. Primer sequences were shown as follows:

IL-1β: F: GACCTGGGCTGTCCTGATG
  R: GAGTGATACTGCCTGCCTGAA
TNFα: F: TCTCATTCCTGCTTGTGG
  R: ACTTGGTGGTTTGCTACG
iNOS: F: CTCGGGTTGAAGTGGTATGC
  R: CCTCCAGGATGTTGTAGCG

#### 4.4.7. RNA-Sequence Assay

The RNA-sequence analysis of macrophage RAW264.7 cells in control and GEP-1 treated groups (200 μg /mL) was performed to explore possible signaling pathways in the immunomodulatory activity of GEP-1. Total RNA was extracted using Trizol reagent (Thermofisher, 15596018) and its quantity and purity were analyzed with Bioanalyzer 2100 and RNA 6000 Nano LabChip Kit (Agilent, CA, USA, 5067-1511). High-quality RNA samples with RIN number >7.0 were used to construct a sequencing library. The mRNA was purified from total RNA (5 μg) using Dynabeads Oligo (dT) (Thermo Fisher Scientific, Inc., Waltham, MA, USA, USA) with two rounds of purification.

A cDNA library constructed by technology from the pooled RNA from macrophage RAW264.7 cells were sequenced and run with the Illumina Novaseq TM 6000 sequence platform. A total of million 2 × 150 bp paired-end Reads was generated through Illumina paired-end RNA-seq approach. To obtain high-quality clean reads, reads were further filtered by Cutadapt (version: cutadapt-1.9). Paired-end clean reads were assigned to the reference genome of the macrophage RAW264.7 cells by using HISAT2 (version:hisat 2-2.2.1) package for further differentially expressed genes (DEGs) analysis. The genes with the parameter of false discovery rate (FDR) below 0.05 and absolute fold change ≥2 were considered differentially expressed genes. Differentially expressed genes were then subjected to enrichment analysis of GO functions and KEGG pathways.

#### 4.4.8. Pathway Assay

The levels of NF-κB and IκB in the macrophage culture were estimated using a rat enzyme-linked immunosorbent assay (ELISA) kit following the manufacturer’s instructions. 

#### 4.4.9. Pathway Inhibition Assay

Cells were pre-treated in the absence or presence of the NF-κB inhibitor PDTC and the IκB inhibitor BAY117082 for 24 h and then incubated with 200 μg/mL of GEP-1 for another 24 h. The levels of TNF-α, IL-1β and NO production in the supernatants were estimated as described above.

### 4.5. Statistical Analysis

Statistical analysis was performed using SPSS 24 software (SPSS Inc., Chicago, IL, USA). Each treatment contains three repetitions, and the average of the three repetitions is taken. Significant differences among treatments were tested with one-way ANOVA at *p* < 0.05 level. The figures of cellular experiments were drawn with GraphPad prism 8.0 (GraphPad Software, San Diego, CA, USA).

## Figures and Tables

**Table 1:** Monosaccharide composition of GEP-1. The undetected monosaccharides are not listed in the table.

Sample	Monosaccharide	Molar Ratio (%)
GEP-1	Ara	2.189
	Gal	4.791
	Glc	92.035
	Man	0.342

**Table 2:** Methylation analysis of GEP-1.

Sample	Linkage	Methylated Sugars	Molecular Weight (MW)	Molar Ratio (%)
	t-Ara(f)	1,4-di-O-acetyl-2,3,5-tri-O-methyl arabinitol	279	0.603
	t-Glc(p)	1,5-di-O-acetyl-2,3,4,6-tetra-O-methyl glucitol	323	7.587
	4-Gal(p)	1,4,5-tri-O-acetyl-2,3,6-tri-O-methyl galactitol	351	0.719
GEP-1	4-Glc(p)	1,4,5-tri-O-acetyl-2,3,6-tri-O-methyl glucitol	351	82.659
	3,4-Glc(p)	1,3,4,5-tetra-O-acetyl-2,6-di-O-methyl glucitol	379	0.520
	2,4-Gal(p)	1,2,4,5-tetra-O-acetyl-3,6-di-O-methyl galactitol	379	0.435
	4,6-Glc(p)	1,4,5,6-tetra-O-acetyl-2,3-di-O-methyl glucitol	379	6.034
	3,6-Gal(p)	1,3,5,6-tetra-O-acetyl-2,4-di-O-methyl galactitol	379	1.444

**Table 3:** Chemical shifts of the main residues of GEP-1.

Code	Residues	Chemical Shifts (ppm)					
		H1/C1	H2/C2	H3/C3	H4/C4	H5/C5	H6/C6
A	⟶4)-α-Glcp-(1⟶	5.33 99.62	3.58 71.91	3.73 73.69	3.61 77.51	3.90 71.66	3.80 60.97
B	⟶4,6)-α-Glcp-(1⟶	5.28 99.75	3.55 71.84	3.67 73.61	3.81 77.34	3.63 71.92	3.37 69.88
C	t-Glcp→	4.91 99.47	3.53 72.71	3.66 72.92	3.32 70.24	3.91 72.37	3.78 60.83

## Data Availability

The data presented in this study are available on request from the corresponding author.

## Sample Availability

Samples of the compounds GEP-1 are available from the authors.
